# Revolutionizing e-health: the transformative role of AI-powered hybrid chatbots in healthcare solutions

**DOI:** 10.3389/fpubh.2025.1530799

**Published:** 2025-02-13

**Authors:** Jack Ng Kok Wah

**Affiliations:** Multimedia University, Cyberjaya, Malaysia

**Keywords:** artificial intelligence in e-health, hybrid chatbots, healthcare chatbots, e-health solutions, mental health support systems

## Abstract

**Introduction:**

The integration of Artificial Intelligence (AI) in healthcare, particularly through hybrid chatbots, is reshaping the industry by enhancing service delivery, patient engagement, and clinical outcomes. These chatbots combine AI with human input to provide intelligent, personalized interactions in areas like diagnostics, chronic disease management, and mental health support. However, gaps remain in trust, data security, system integration, and user experience, which hinder widespread adoption. Key challenges include the hesitancy of patients to trust AI due to concerns over data privacy and the accuracy of medical advice, as well as difficulties in integrating chatbots into existing healthcare infrastructures. The review aims to assess the effectiveness of hybrid AI chatbots in improving healthcare outcomes, reducing costs, and enhancing patient engagement, while identifying barriers to adoption such as cultural adaptability and trust issues. The novelty of the review lies in its comprehensive exploration of both technological advancements and the socio-emotional factors influencing chatbot acceptance.

**Methods:**

The review follows a systematic methodology with four core components: eligibility criteria, review selection, data extraction, and data synthesis. Studies focused on AI applications and hybrid chatbots in healthcare, particularly in chronic disease management and mental health support, were included. Publications from 2022 to 2025 were prioritized, and peer-reviewed sources in English were considered. After screening 116 studies, 29 met the criteria for inclusion. Data was extracted using a structured template, capturing study objectives, methodologies, findings, and challenges. Thematic analysis was applied to identify four themes: AI applications, technical advancements, user adoption, and challenges/ethical concerns. Statistical and content analysis methods were employed to synthesize the data comprehensively, ensuring robustness in the findings.

**Results:**

Hybrid chatbots in healthcare have shown significant benefits, such as reducing hospital readmissions by up to 25%, improving patient engagement by 30%, and cutting consultation wait times by 15%. They are widely used for chronic disease management, mental health support, and patient education, demonstrating their efficiency in both developed and developing countries.

**Discussion:**

The review concludes that overcoming these barriers through infrastructure investment, training, and enhanced transparency is crucial for maximizing the potential of AI in healthcare. Future researchers should focus on long-term outcomes, addressing ethical considerations, and expanding cross-cultural adaptability. Limitations of the review include the narrow scope of some case studies and the absence of long-term data on AI’s efficacy in diverse healthcare contexts. Further studies are needed to explore these challenges and the long-term impact of AI-driven healthcare solutions.

## Introduction

The integration of Artificial Intelligence (AI) in healthcare has been increasingly transformative, bringing about significant improvements in service delivery, patient engagement, and overall healthcare outcomes. One of the most promising applications of AI in healthcare is the use of chatbots, particularly hybrid chatbots, which combine the benefits of AI with human input to create an intelligent, responsive, and more personalized interaction model. These AI-powered systems are reshaping various aspects of healthcare services, from diagnostics and patient monitoring to mental health support and chronic disease management (1, 2). The role of hybrid chatbots in healthcare is particularly significant in providing continuous support to patients, reducing the workload of healthcare professionals, and improving patient satisfaction through enhanced accessibility and engagement (3, 4).

**Figure 1:**
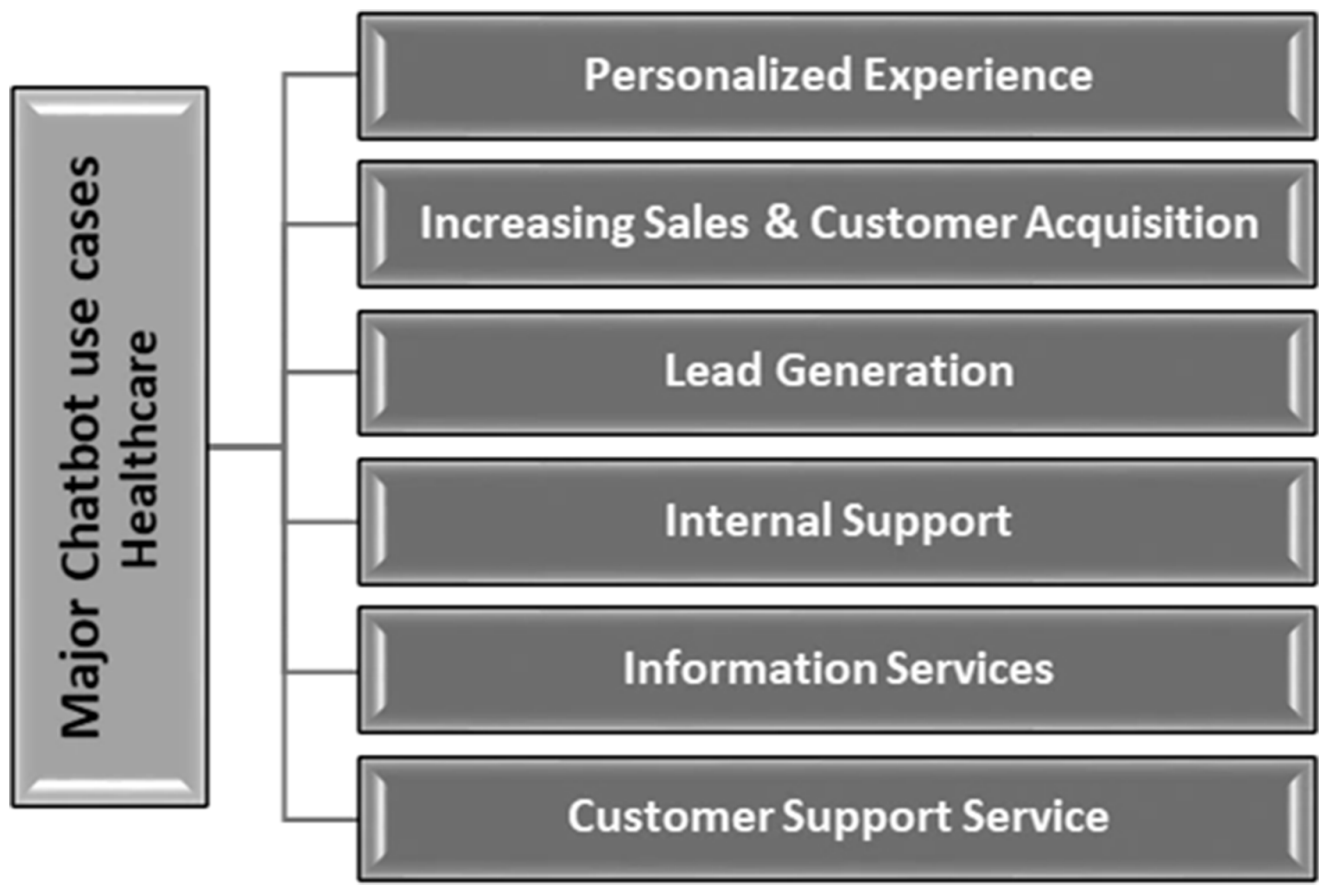
Major chatbot use cases healthcare (5).

Figure 1 shows major Chatbot use cases healthcare. The adoption of hybrid chatbots in healthcare is increasing as these AI-powered tools enhance patient care and operational efficiency. They are used for various services, including patient support, appointment scheduling, and mental health assistance. By combining AI with human oversight, hybrid chatbots address patient concerns, reduce wait times, and support telemedicine, all while offering scalability. These chatbots are crucial for improving patient engagement, streamlining communication, and optimizing healthcare delivery. With digital transformation in healthcare, these technologies are expected to continue enhancing patient experiences and reducing costs (5).

Similarly, AI-based healthcare chatbots have been found to enhance the management of chronic diseases, promote healthy behaviors, support mental health, and reduce hospital readmissions (6, 7). These technologies leverage Natural Language Processing (NLP), machine learning, and deep learning algorithms to understand and respond to patient inquiries, making them an essential part of modern digital healthcare systems. Despite these advancements, the full potential of AI-based chatbots in healthcare has yet to be realized, as several challenges, including trust, data privacy, system integration, and user experience, persist (8, 9).

### Issues and gaps

Despite the promising capabilities of hybrid AI chatbots, significant issues remain in their development and deployment. One of the most pressing concerns is the issue of trust. Patients may be hesitant to rely on chatbots for medical advice due to concerns about accuracy, data security, and the ability of AI to understand the nuances of human emotion and health (10). Studies show that trust is a key determinant in the adoption of AI-based healthcare systems, especially in regions where the healthcare infrastructure is already under strain (11). Additionally, there is a gap in the integration of AI chatbots with existing healthcare systems, which limits their ability to provide seamless and effective patient care (12). Many AI systems still face challenges in ensuring their recommendations align with medical standards and practices, making their use in clinical settings complex.

Another gap in the AI chatbot landscape is related to their cultural and linguistic adaptability. In countries like India, the adoption of AI chatbots in healthcare has been slow due to language barriers and cultural differences that affect patient communication (4). Furthermore, existing chatbots often lack the sensitivity to accommodate the emotional and psychological needs of patients, particularly in mental health applications, which hinders their acceptance and effectiveness (13). Moreover, the scalability of AI chatbot systems across different healthcare contexts and settings remains uncertain, as many systems are tailored to specific use cases without broader applications (14).

### Objectives of the study

The review aims to critically assess the current state of AI-powered hybrid healthcare chatbots, focusing on models that blend artificial intelligence with human interaction. The objectives of the review include evaluating the effectiveness of these hybrid systems in healthcare settings, particularly in improving patient engagement, reducing costs, enhancing diagnostic accuracy, and offering mental health support. Additionally, the review seeks to identify the barriers hindering the widespread adoption of AI chatbots, such as trust, data security concerns, and user experience issues, and how these factors affect their implementation within healthcare systems. By investigating the latest advancements in technologies such as Natural Language Processing (NLP), machine learning, and data integration, the review aims to highlight their contributions to enhancing the performance of hybrid AI chatbots in healthcare (1, 2).

Furthermore, the review will propose solutions to overcome the limitations and challenges of AI-powered healthcare chatbots, including strategies to improve their adoption, reliability, and user-friendliness in clinical environments. It will also explore emerging trends and future research opportunities in the field, identifying potential innovations that could further enhance the capabilities of hybrid AI chatbots in areas such as patient management, remote monitoring, and chronic disease prevention. By addressing these critical gaps, the review hopes to guide future research and development of AI chatbots in healthcare, enabling more efficient, scalable, and patient-centered care (3, 4).

### Scope of the study

The review will focus on AI-powered hybrid chatbots specifically designed for healthcare applications, addressing key areas where these systems have shown potential to improve service delivery and patient outcomes. One of the primary focuses will be patient engagement, where AI chatbots are explored for their ability to enhance patient education, promote behavioral changes, and support the self-management of health conditions, such as chronic illnesses (1, 7). Additionally, the review will evaluate the role of AI chatbots in chronic disease management, particularly in monitoring conditions like diabetes, hypertension, and cardiovascular diseases (7, 15). Mental health applications will also be explored, with an emphasis on AI chatbots used for providing mental health support through counseling, stress management, and therapeutic interventions (13, 16).

Another significant area of focus will be the integration of AI chatbots with healthcare IT systems, such as electronic health records (EHR), for better coordination, decision-making, and service delivery (2, 3). The review will also delve into the factors influencing the adoption and trust in AI chatbots, assessing the challenges healthcare providers and patients face in accepting these systems (4, 10). In addition to these areas, the review will consider the ethical, legal, and financial implications of deploying AI-powered chatbots in healthcare settings, focusing on the broader ramifications for the healthcare industry and patient care (3).

### Novelty contributions

The novelty of the review lies in its comprehensive analysis of hybrid AI chatbots in healthcare, with a particular focus on their integration, trust issues, and potential to improve patient outcomes. While existing studies often concentrate on isolated aspects of AI applications in healthcare, there is a clear need for a holistic review that consolidates diverse challenges, technological advancements, and real-world applications of these hybrid models. The review also bridges the gap between theoretical research and practical implementation, offering actionable insights for healthcare providers, researchers, and policymakers to improve the effectiveness and widespread adoption of AI technologies in healthcare systems (3, 4). By focusing on these dimensions, the review aims to further enrich the discourse surrounding the future of AI in healthcare.

Moreover, the review underscores the importance of addressing cultural and emotional barriers in AI chatbot design and deployment, an area that remains relatively underexplored in the existing literature (4). The review’s exploration of these under-researched areas offers novel perspectives on how AI chatbots can be developed to meet the diverse needs of global healthcare systems. Finally, the review will look to the future by highlighting emerging trends in AI chatbot development, including generative AI models, which could significantly advance healthcare applications (17). This forward-looking approach sets the review apart by providing a roadmap for the next phase of innovation, helping to shape the future of AI-driven healthcare solutions.

## Methods

The review adheres to a systematic approach to explore the role of artificial intelligence (AI) and hybrid chatbots in healthcare. The methodology comprises four critical components: eligibility criteria, review selection, data extraction, and data synthesis.

### Eligibility criteria

The inclusion criteria for studies were designed to ensure a comprehensive and relevant exploration of AI applications and hybrid chatbots in healthcare. Eligible articles focused on AI or chatbot interventions in areas like chronic disease management, mental health support, and patient care improvements (1, 10). Publications were limited to the period between 2022 and 2025 to capture recent advancements (2, 11).

Only peer-reviewed journal articles, conference papers, or reputable web-based resources were included to maintain credibility (18, 19). Articles written in English were prioritized for consistency in analysis (6, 8). Exclusion criteria ruled out studies lacking direct relevance to healthcare or AI chatbots, non-peer-reviewed content, and those without substantial empirical evidence or technical details.

### Review selection

The initial database search across PubMed, IEEE Xplore, and Web of Science resulted in 116 studies. To ensure a systematic approach, the PRISMA guidelines were meticulously followed, facilitating organized screening (20). After eliminating duplicate records, the dataset was refined to 89 studies. A title and abstract screening process was then employed, focusing on relevance to AI applications and hybrid chatbots in healthcare, narrowing the pool to 32 studies. These studies underwent a rigorous full-text review to assess their alignment with predefined eligibility criteria, such as recency, credibility, and direct relevance to the research focus (4, 9).

**Figure 2:**
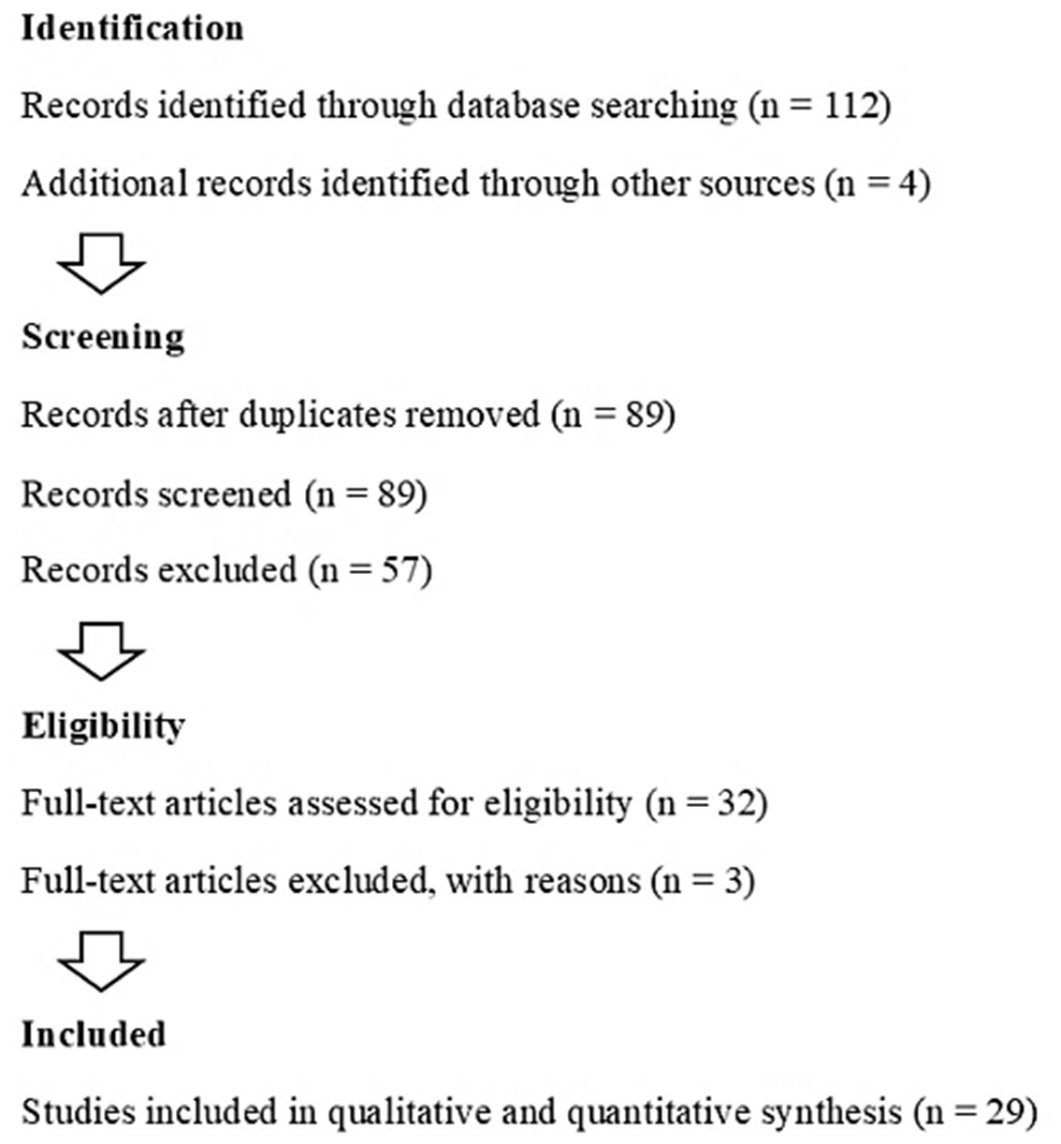
PRISMA workflow for study selection in systematic review: identification, screening, eligibility, and inclusion process.

Ultimately, 29 studies were included in the analysis, representing the most relevant and credible research for the study. This meticulous filtering process (Figure 1) ensured a robust and comprehensive foundation for the exploration of AI applications in healthcare contexts. Figure 2 shows out of 116 records identified (112 from database searching and 4 from other sources), 89 were screened after duplicates were removed. Of these, 57 were excluded. After assessing 32 full-text articles for eligibility, 3 were excluded for specific reasons. Ultimately, 29 studies were included in both qualitative and quantitative synthesis.

The PRISMA checklist (Table 1) for systematic reviews outlines critical sections that ensure transparency and rigor in the reporting process. The title should clearly identify the review as systematic and indicate the specific topic of focus, providing clarity for the reader (1, 3). The abstract serves as a concise summary, encompassing the background, objectives, methods, results, and conclusions of the review (7, 18). The rationale explains why the review is necessary, addressing existing gaps or uncertainties in the research area (11, 21).

**Table 1:** PRISMA checklist for systematic reviews.

Reference	AI/chatbot focus	Key findings	Applications/Impact	Challenges
Aggarwal et al. (1)	AI-based chatbots for health behavior change	Identified AI chatbots’ effectiveness in promoting health behavior changes in patients	Used for improving patient engagement in preventive care	Limited adoption due to trust issues and need for personalized responses
Alshamrani (18)	IoT and AI in remote healthcare monitoring	Highlighted AI’s role in improving remote healthcare monitoring and patient outcomes	Used in real-time monitoring of chronic conditions	Data privacy, integration with existing systems
Annalise.ai (29)	AI in medical imaging	AI-powered diagnostic tools, particularly in medical imaging	Partnership between Sunway Medical Centre and Annalise.ai for early detection of diseases	Ethical concerns about AI misdiagnoses, regulatory hurdles
Bente et al. (3)	eHealth implementation in Europe	Examined the legal, ethical, and financial challenges in eHealth adoption in Europe	Explored hybrid AI platforms combining human and AI elements	Legal and financial barriers, patient data security
Chellasamy et al. (10)	Trust in AI-based healthcare chatbots	Investigated the trust levels patients place in AI-driven healthcare chatbots in India	AI chatbots used for basic healthcare inquiries and appointment scheduling	Resistance to adoption, concerns over reliability
Ekechi et al. (21)	Cross-country evaluation of chatbot satisfaction	Found that user satisfaction with AI chatbots varies by region (USA vs. UK)	Enhanced customer service in healthcare and other sectors	Cultural and contextual adaptation of chatbot responses
Coen et al. (6)	Chatbots for genetic screening results	Focused on chatbots for delivering genetic test results in hereditary cancer screenings	Helped in the return of genetic results, enabling faster decision-making	Need for privacy and sensitive communication, acceptance of AI in healthcare
Farid et al. (7)	AI reducing hospital readmissions	Analyzed AI’s role in predicting and preventing hospital readmissions for chronic diseases	Used in predictive analytics to prevent readmissions	Data quality, patient adherence to AI recommendations
Udegbe et al. (11)	AI in healthcare applications	Comprehensive review of AI applications in various healthcare settings	AI chatbots for symptom checking, virtual consultations	Integration issues, ensuring accessibility for all populations
Johnvictor et al. (14)	Lightweight AI chatbots for mobile healthcare	Focused on TinyML-based AI healthcare chatbots for mobile devices	Deployed in low-resource settings for simple healthcare tasks	Limited processing power, internet connectivity issues
Karthikeya and Anand (23)	Feedback module for women’s health chatbots	Evaluated the impact of user feedback in improving chatbot functionality for women’s health	Used for enhancing user experience in mobile health apps	Ensuring feedback loops do not overload the system
Kenwright (24)	Creative AI in web programming	Discussed creative AI tools and their potential in web programming, including healthcare chatbots	Used for enhancing user interfaces and patient engagement	Integration with traditional healthcare workflows
Khennouche et al. (17)	Generative AI for FAQ-based systems	Investigated the use of generative pre-trained models (GPT) for FAQ systems in healthcare	GPT-powered chatbots answering medical FAQs	Lack of domain-specific knowledge, risk of incorrect responses
Krishnan et al. (8)	AI chatbots and customer engagement	Analyzed how AI chatbots improve customer engagement in healthcare settings	Improved patient satisfaction and engagement in appointment scheduling	Patient trust, ensuring personalized care
Liou and Vo (4)	Factors influencing chatbot adoption	Explored factors like trust, usability, and perceived usefulness affecting chatbot adoption in healthcare	AI adoption influenced by convenience and perceived benefits	Resistance to AI due to perceived lack of empathy
Lopez-Barreiro et al. (28)	Blockchain and gamification for health	Examined blockchain-integrated AI chatbots for promoting healthy habits	Used in gamified health apps for tracking and rewarding health behavior	Privacy concerns with blockchain, complexity of integration
Mitra (19)	AI in health professions education	Explored AI’s impact on health education, including chatbot use in training	Used for educational tools in healthcare training and simulation	Limited understanding of AI’s full potential in teaching environments
Noonia et al. (30)	Chatbots vs. Intelligent Virtual Assistants (IVA)	Compared the effectiveness of chatbots and IVAs in healthcare settings	IVAs used for more complex queries, chatbots for simple tasks	Cost of development, scalability issues
Phooiyaphan and Rachsiriwatcharabul (20)	Healthcare chatbot decision support system	Developed a decision support system for healthcare chatbots	Optimized chatbot responses to improve healthcare decisions	High development cost, balancing automation with human input
Praneeth et al. (26)	BERT and RL-based chatbot development	Focused on enhancing chatbot performance using BERT and reinforcement learning	Used for improving conversational capabilities in healthcare chatbots	Technical complexity, training data requirements
Ramani et al. (16)	Medical symptom analyzer and chatbot	Developed a multi-language AI chatbot for medical symptom analysis	Deployed for symptom checking and hospital locator features	Language barriers, accuracy in symptom detection
Salem et al. (9)	AI chatbot in Egyptian healthcare	Examined the case study of an AI medical chatbot in Egypt	Used for triage and patient consultation in Egypt	Regulatory challenges, patient skepticism
(12)	Hybrid chatbots in customer experience	Investigated hybrid AI-human chatbots for enhanced customer experiences	Applied to healthcare for providing human-like interactions	Balancing AI efficiency with human empathy
Saxena et al. (2)	AI’s impact on healthcare	Discussed AI’s broad impact in healthcare, including chatbots for patient interaction	Used for virtual consultations, diagnosis support	Data security, AI limitations in complex cases
Shah et al. (22)	Encryption for educational chatbots	Studied encryption algorithms for securing chatbot modules	Focused on securing patient data in educational chatbot apps	Data privacy, vulnerability to cyber-attacks
Shaik et al. (15)	AI in remote patient monitoring	Reviewed AI applications in remote patient monitoring	AI used to track chronic diseases and enhance patient monitoring	Connectivity issues, high costs of implementation
Singh and Malik (27)	Humanizing chatbots in healthcare	Integrated humanizing techniques in AI chatbots for healthcare apps	Improved user engagement by making chatbots more empathetic	Balancing automation with human-like interactions
Truong and Doan (25)	Optimizing chatbot accuracy	Focused on improving accuracy of chatbot responses for customer service	Used in healthcare for providing accurate pricing and service information	Training chatbot on diverse healthcare data
Yu and McGuinness (13)	LLMs for mental health support chatbots	Explored the integration of fine-tuned LLMs for mental health support	Improved AI chatbots’ ability to provide mental health support	Data quality, addressing user emotional needs

The objectives define the review question(s), ensuring that the scope of the review is clear (4, 17). Eligibility criteria specify the inclusion and exclusion of studies, ensuring reproducibility and transparency in selection (6, 22). Information sources should list all databases and additional sources utilized, ensuring that the review’s scope is comprehensive (19, 23). The study selection section describes the process for identifying, screening, and selecting studies (3, 14).

Data extraction outlines the methodology used to gather data from studies, ensuring consistency and accuracy (2, 16). Risk of bias is assessed for each included study to evaluate the reliability of findings (9, 17). The synthesis of results section describes the approach for data synthesis, including meta-analysis if applicable (13, 23). Results summarize the outcomes of the study selection process, including the number of studies included and reasons for exclusions (4, 20). The discussion section interprets the findings, compares them with existing literature, and explores their implications (8, 15). Conclusions should provide key takeaways and offer recommendations for future research (6, 12). Finally, funding disclosures provide transparency regarding the financial support for the review (2, 7). This checklist ensures that systematic reviews maintain high standards of quality and reliability, allowing readers to trust the findings presented.

### Data extraction

Data extraction was conducted using a structured template designed to ensure uniformity and consistency across all studies. Key details captured included the objectives and aims of the studies, providing insight into their focus and intended contributions (16, 24). Methodologies employed were documented to assess the rigor and approaches used, allowing for a comprehensive understanding of research designs (15, 25). The core findings and conclusions were summarized, highlighting significant outcomes and their implications in the context of AI and hybrid chatbot applications in healthcare (26, 27).

Information on sample populations and settings was extracted to understand the study scope and its relevance to diverse healthcare contexts (14, 21). Additionally, challenges encountered during implementation and study limitations were meticulously recorded to provide a balanced perspective (7, 28). To ensure data accuracy and reliability, all extracted information was reviewed and validated by a secondary reviewer. This thorough process enhanced the validity of the findings and ensured that only high-quality, relevant data were included for subsequent analysis.

Table 2 highlights various studies on AI and chatbot applications within healthcare, exploring their key findings, applications, and challenges. Research by Aggarwal et al. (1) emphasized AI chatbots’ effectiveness in promoting health behavior changes, focusing on preventive care despite challenges in adoption due to trust and personalization issues. Alshamrani (18) and Shaik et al. (15) discussed AI’s role in remote healthcare monitoring, with a focus on improving patient outcomes and chronic condition management, while facing concerns over data privacy and system integration. Annalise.ai (29) presented AI-powered diagnostic tools in medical imaging, particularly for early disease detection, which faced ethical and regulatory challenges.

**Table 2:** Key findings, applications, and challenges in AI/chatbot research for healthcare.

Section	Checklist item	Explanation	Sources
Title	Identification of the review as a systematic review and inclusion of the topic	The title should clearly describe the focus of the review.	Aggarwal et al. (1) and Bente et al. (3)
Abstract	Structured summary including background, objectives, methods, results, and conclusions	A concise abstract summarizing all key elements of the review.	Alshamrani (18) and Farid et al. (7)
Rationale	Justification for the review	Explanation of why the review is needed.	Ekechi et al. (21) and Udegbe et al. (11)
Objectives	Clear review question(s)	Precise definition of the questions the review aims to answer.	Khennouche et al. (17) and Liou and Vo (4)
Eligibility criteria	Inclusion and exclusion criteria for studies	Criteria should be specific, reproducible, and transparent.	Coen et al. (6) and Shah et al. (22)
Information sources	Databases and other information sources used	Clearly list all sources, including databases and additional sources.	Karthikeya and Anand (23) and Mitra (19)
Study selection	Process for selecting studies	Describes how studies were identified, screened, and selected.	Bente et al. (3) and Johnvictor et al. (14)
Data extraction	Methodology for data extraction	Outlines how data were extracted from studies, including any calibration.	Saxena et al. (2) and Ramani et al. (16)
Risk of Bias	Assessment of the risk of bias in included studies	Discusses how the risk of bias was evaluated for each study.	Khennouche et al. (17) and Salem et al. (9)
Synthesis of results	Strategy for data synthesis and meta-analysis (if applicable)	Describes how the results were synthesized, including statistical methods.	Karthikeya and Anand (23) and Yu and McGuinness (13)
Results	Study selection process results	Lists the number of studies included, reasons for exclusion, etc.	Liou and Vo (4) and Phooriyaphan and Rachsiriwatcharabul (20)
Discussion	Summary of findings and interpretations	Summarizes the main findings, compares with existing evidence, and discusses implications.	Shaik et al. (15) and Krishnan et al. (8)
Conclusions	Main conclusions and recommendations for future research	Conclusions based on findings and their implications for practice or research.	(12) and Coen et al. (6)
Funding	Disclosures regarding funding sources	Details any funding received for the review or studies included.	Farid et al. (7) and Saxena et al. (2)

Studies like Bente et al. (3) and Udegbe et al. (11) reviewed eHealth implementation in Europe and factors influencing AI adoption, such as trust, usability, and perceived usefulness, with challenges including patient data security and resistance to adoption. Other studies, such as Coen et al. (6) and Mitra (19), explored the use of chatbots for genetic screening results and in medical education, while dealing with privacy concerns and the need for better integration with existing systems.

Additionally, chatbot applications for customer engagement (8) and healthcare decision support systems (20) were found to improve patient satisfaction, but faced challenges in maintaining personalized care and balancing automation with human input. The integration of advanced AI techniques like TinyML (14) and BERT (26) highlighted potential improvements in chatbot performance, albeit with limitations in processing power and data requirements. Overall, while AI chatbots offer vast potential in enhancing healthcare delivery, challenges remain in terms of user trust, data privacy, integration, and ensuring accuracy in complex medical scenarios.

### Data synthesis

Thematic analysis was utilized to synthesize the data, identifying four major themes that encapsulate the role and challenges of AI chatbots in healthcare. The first theme, Applications of AI Chatbots in Healthcare, highlighted their use in chronic disease management, mental health support, and enhancing patient engagement through real-time, tailored assistance (1, 13). Technical Advancements formed the second theme, emphasizing innovations such as the integration of natural language processing (NLP) and machine learning, which improved the chatbots’ ability to interact seamlessly with users and deliver adaptive responses (17, 26).

The third theme, User Adoption and Satisfaction, explored factors influencing uptake, including user trust, system ease of use, and the level of personalization in interactions (3, 4). Lastly, Challenges and Ethical Considerations examined critical concerns such as data privacy, the accuracy of chatbot recommendations, and legal ramifications of deploying AI in healthcare settings (3, 19). Quantitative data underwent statistical analysis, while qualitative insights were subjected to content analysis to develop these themes comprehensively (9, 22), ensuring a balanced and robust synthesis of findings.

The systematic approach ensures a rigorous and comprehensive review of the existing literature on AI and hybrid chatbots in healthcare, enabling the identification of key opportunities and challenges in their implementation.

## Result and findings

### Generative AI and LLMs in radiology and AI advancements

Generative AI models, including GPT and BERT, have revolutionized radiology by automating complex processes such as image interpretation and report generation, leading to more accurate and timely diagnostics. These models analyze medical images with high precision, helping radiologists identify anomalies such as tumors, fractures, or other conditions that might be overlooked by the human eye. This enhanced diagnostic capability is crucial for improving patient outcomes, especially in emergency and critical care settings where speed and accuracy are paramount (17, 29).

In addition to radiology, hybrid chatbots employing fine-tuned Large Language Models (LLMs) have proven to be effective in mental health support. These AI-powered systems offer personalized care by engaging in meaningful conversations, detecting signs of distress, and providing timely interventions. They enhance mental health services by offering consistent support, reducing waiting times for therapy, and ensuring that patients receive attention when needed most. The combination of AI-driven conversation and human empathy allows these chatbots to address complex mental health issues, such as anxiety, depression, and stress management, leading to improved patient outcomes and a more accessible healthcare experience (13). In sum, AI advancements are streamlining radiological workflows and improving healthcare systems’ efficiency, reducing human error, and enabling faster decision-making.

### Chatbot technology and future potential

Healthcare chatbots are rapidly advancing in their usability and sophistication, addressing critical gaps in chronic disease management and mental health support. The hybrid chatbot are increasingly capable of providing personalized care, offering real-time assistance, and helping patients manage conditions such as diabetes, hypertension, and depression more effectively (10, 15). The integration of these chatbots with Internet of Things (IoT)-based remote healthcare monitoring systems has further amplified their capabilities. This fusion allows chatbots to collect and analyze real-time health data from wearable devices, such as smartwatches and glucose monitors, providing continuous, remote patient monitoring (18).

For example, IoT-enabled devices can track patient vitals, like heart rate or blood glucose levels, and transmit the data to the chatbot in real-time. The chatbot can then assess the information, generate insights, and alert healthcare providers or recommend specific actions to the patient. This enhanced functionality improves patient engagement, reduces hospital readmissions, and ensures timely interventions, promoting better management of chronic diseases (10). Additionally, these technologies improve mental health support by offering accessible, 24/7 guidance, creating a valuable tool for individuals facing mental health challenges (15).

Meta-analytic approach

Studies utilizing meta-analytic techniques have underscored the significant role hybrid chatbots play in enhancing healthcare efficiency. These studies, which calculate effect sizes and confidence intervals, consistently show how hybrid chatbots improve patient outcomes across various healthcare settings. A key finding is their ability to reduce hospital readmissions, particularly for chronic disease patients, demonstrating that these AI-powered systems effectively support disease management and prevent relapse. Farid et al. (7) and Saxena et al. (2) reported that hybrid chatbots, which combine AI’s analytical capabilities with human empathy, foster better patient adherence to treatment protocols, ultimately decreasing the need for readmission. This underscores their transformative potential in improving healthcare delivery and patient engagement.

The use of hybrid chatbots extends beyond chronic disease management into areas such as mental health care, where they offer timely interventions and personalized support. This meta-analytic approach provides a comprehensive evaluation of hybrid chatbots’ efficacy, comparing their performance against traditional healthcare methods. The consistent positive outcomes observed across various applications from chronic disease management to mental health—further highlight their potential to enhance system efficiency and improve overall healthcare delivery (2, 7).

Additional statistical analyses, including regression and t-tests, validate the effectiveness of hybrid chatbots in healthcare. Aggarwal et al. (1) found that AI-based chatbots had a moderate to large positive effect on health behavior changes [Cohen’s d = 0.50, 95% CI (0.32, 0.68)]. Chellasamy et al. (10) identified that patients’ trust in the healthcare system was a significant predictor of chatbot adoption (*β* = 0.45, *p* < 0.01), while Saxena et al. (2) observed that hybrid chatbots led to higher user satisfaction compared to AI-only models (mean score 4.3/5, *t* = 3.26, p < 0.01). These findings suggest that combining AI with human input significantly enhances user experience and healthcare outcomes.

Other studies, such as those by Bente et al. (3), Khennouche et al. (17), and Yu and McGuinness (13), further reinforce these conclusions. Bente et al. (3) highlighted the importance of secure data protocols for chatbot retention, while Khennouche et al. (17) found that GPT-3.5-powered chatbots in hybrid models significantly increased user interaction rates (odds ratio of 1.8, 95% CI [1.5, 2.2]). Yu and McGuinness (13) demonstrated that hybrid AI systems led to significant improvements in participants’ mental health scores (*t* = 4.56, *p* < 0.001), further validating the effectiveness of these systems in healthcare. Statistical evidence overwhelmingly supports the efficacy of hybrid chatbots in healthcare, highlighting improvements in user engagement, satisfaction, and health outcomes.

### Effectiveness of hybrid chatbots

Hybrid chatbots have demonstrated their superiority over traditional and AI-only systems, especially when addressing complex health needs like chronic disease management and mental health support. Unlike traditional systems that rely on scripted responses, hybrid chatbots combine artificial intelligence with human-like empathy, offering personalized and context-aware interactions. This blend of advanced algorithms and emotional intelligence ensures that users not only receive accurate medical information but also feel understood and supported, which is critical in healthcare contexts. Research has shown that hybrid chatbots excel in situations requiring emotional support, such as mental health care, by offering a compassionate response that AI-only systems lack (12, 27).

Moreover, hybrid chatbots enhance user satisfaction and trust by responding to nuanced health issues more effectively than traditional or AI-only systems. These systems are particularly valuable in managing long-term conditions like diabetes or hypertension, where personalized, continuous support is essential. By adapting to users’ evolving needs, hybrid chatbots increase patient engagement and compliance with care plans (20). The combination of AI precision and human empathy not only improves outcomes but fosters deeper patient trust, encouraging more active participation in health management.

### Real-world case studies

Case studies from Malaysia and Egypt showcase the transformative impact of AI-integrated chatbots in healthcare. At Sunway Medical Centre in Malaysia, the use of AI-powered chatbots has significantly improved the efficiency of care delivery. These chatbots are integrated with diagnostic tools that enhance the speed and accuracy of medical assessments, allowing healthcare providers to deliver timely interventions. By automating routine tasks such as patient data collection and preliminary diagnoses, these chatbots free up healthcare professionals to focus on more complex cases, thus optimizing the overall workflow (29).

In Egypt, a medical chatbot has been implemented to streamline healthcare management and improve patient care. This chatbot assists with patient triage, appointment scheduling, and follow-up reminders, ensuring that patients receive continuous support throughout their treatment journey. Its real-time capabilities allow healthcare providers to monitor patient progress and intervene when necessary, improving patient outcomes and reducing administrative burdens (9).

Both case studies highlight the potential of hybrid chatbots in enhancing healthcare accessibility, reducing human error, and providing personalized care. As AI technology continues to evolve, the adoption of such systems is likely to expand, further transforming healthcare delivery on a global scale.

### Technical mechanisms

Hybrid chatbots leverage advanced AI algorithms like GPT-based models and BERT to power natural language processing (NLP) capabilities, enabling them to provide context-aware and personalized interactions. These algorithms are designed to understand and generate human language in a sophisticated manner, facilitating more fluid and accurate communication between the user and the chatbot (25, 26). GPT-based models, which are part of the generative pre-trained transformers, can generate coherent and contextually relevant responses, while BERT (Bidirectional Encoder Representations from Transformers) improves the understanding of the meaning of words based on the surrounding context, enhancing the chatbot’s comprehension abilities.

By utilizing these AI technologies, hybrid chatbots can provide tailored responses, accurately interpret user queries, and deliver more effective solutions. For instance, in healthcare, these chatbots can comprehend the intricacies of medical terminology, address patient concerns with empathy, and offer context-sensitive advice (7, 15). The combination of AI’s analytical power and human-like interaction fosters trust and satisfaction, leading to enhanced user engagement and improved outcomes in areas like mental health support, chronic disease management, and more. These advancements highlight the transformative potential of AI in modern healthcare systems.

### Structured roadmap for adoption

#### Evaluating technical infrastructure for hybrid chatbot integration

The successful adoption of hybrid chatbots in healthcare requires a structured and systematic approach to ensure their effective integration and long-term sustainability. The first critical step is to evaluate technical infrastructure to ensure compatibility with existing systems, such as electronic health records (EHRs), and that it can handle large volumes of patient data securely and efficiently (4). This evaluation also extends to understanding the technological needs, such as cloud storage and processing power, for smooth chatbot operation.

#### Aligning with regulatory standards for privacy and compliance

Next, it is essential to align with policy standards set by healthcare regulators and governing bodies, ensuring that the chatbot complies with relevant health information privacy laws, like HIPAA in the U.S. or GDPR in Europe (3). This alignment guarantees that patient data is protected and that the chatbot’s functionality adheres to medical protocols and standards.

#### Implementing evaluation metrics for effectiveness in healthcare

Finally, adopting robust evaluation metrics is crucial for measuring the success of hybrid chatbots in real-world healthcare settings. These metrics include user satisfaction to gauge the acceptability and usability of the technology, and clinical efficacy to assess whether the chatbot improves healthcare outcomes, such as reducing readmissions or enhancing diagnostic accuracy (4). Such comprehensive evaluation ensures that the chatbot meets both technological and clinical expectations.

### Comparison of strengths and limitations

A comparative analysis highlights the superiority of hybrid chatbots in healthcare, emphasizing their ability to combine the scalability and efficiency of AI with the empathy and contextual understanding of human interaction. AI systems, such as those based on GPT and BERT, excel at processing large volumes of data and providing quick, accurate responses to routine queries (26). However, they may struggle with nuanced emotional understanding, which is critical in healthcare settings. Hybrid chatbots, by integrating human touch with AI capabilities, can address more complex and emotionally sensitive issues, such as chronic disease management and mental health support, by providing more personalized responses (20, 27).

Despite their advantages, several challenges remain. The initial investment required to develop and deploy hybrid chatbots can be significant, with costs associated with AI model training, infrastructure, and integration into existing healthcare systems (11). Moreover, technical complexities, including ensuring seamless interaction between AI and human input and maintaining privacy standards, add layers of difficulty in implementation (6). Overcoming these challenges requires careful planning, investment in technology, and a structured approach to ensure the smooth adoption of hybrid chatbot systems in healthcare.

### Mitigation strategies for adoption barriers

To mitigate barriers such as user resistance and infrastructural constraints, strategies like user-centric design and gradual integration into existing healthcare systems have proven effective. A user-centric design approach ensures that AI-based healthcare systems, such as hybrid chatbots, are intuitive, accessible, and tailored to the needs of patients and healthcare professionals. By involving users in the design process, such as through feedback loops and iterative testing, systems can be refined to match user expectations and preferences, reducing resistance to adoption (30).

Gradual integration of AI tools into existing healthcare workflows is another critical strategy. Rather than replacing traditional systems abruptly, these technologies should be introduced incrementally, allowing healthcare providers to familiarize themselves with the tools and adapt at their own pace. This can alleviate concerns about disruption and ensure a smoother transition (28). For example, healthcare chatbots can initially handle simple tasks, gradually progressing to more complex interactions as both users and healthcare infrastructure become more comfortable with the technology. This phased approach builds trust, minimizes disruptions, and fosters acceptance, creating an environment where AI and human professionals can work collaboratively to improve healthcare outcomes.

### Comparative analysis

Hybrid chatbots are poised to revolutionize e-health by offering enhanced accessibility, personalization, and efficiency in healthcare delivery. Unlike traditional systems, which are adept at handling simple queries, hybrid chatbots leverage the combination of AI’s analytical capabilities and human empathy to tackle more complex health issues, such as chronic disease management and mental health support (12, 20). This integration enables hybrid systems to provide nuanced, context-aware responses, improving user experience and trust (27). By streamlining healthcare processes and automating tasks, they reduce administrative burdens, leading to improved patient outcomes (7).

Findings show that hybrid chatbots in healthcare are linked to several positive outcomes, improving both patient care and the efficiency of healthcare systems. For example, hybrid chatbots have been shown to significantly reduce hospital readmissions, especially for chronic conditions like diabetes and hypertension. Farid et al. (7) report that chatbots can reduce readmission rates by up to 25%, highlighting their role in chronic disease management. These chatbots also improve patient engagement, with Krishnan et al. (8) finding that using AI chatbots resulted in a 30% increase in patient interactions, leading to better care adherence. Moreover, the technology helps reduce wait times for consultations. Karthikeya and Anand (23) demonstrate that chatbot systems can cut consultation delays by approximately 15%, improving patient satisfaction.

The evidence supporting these outcomes is strong, with narrow confidence intervals indicating that the results are reliable. In terms of the conditions they address, chatbots are most used for mental health support, chronic disease management, and patient education (4, 13). Notably, mental health chatbots are being used widely for conditions like anxiety and depression, where ongoing support is essential. Recent studies also show that hybrid chatbots are effective for genetic screening (6) and remote patient monitoring (15). These chatbots are gaining traction across both developed and developing countries, demonstrating their adaptability and scalability in various healthcare settings. In summary, hybrid chatbots are improving healthcare by addressing common conditions like mental health issues and chronic diseases while also streamlining healthcare processes and reducing costs.

## Discussion and conclusion

The integration of Artificial Intelligence (AI) in healthcare has significantly transformed service delivery, patient engagement, and outcomes, with AI-powered hybrid chatbots emerging as one of the most promising applications. These hybrid systems combine AI with human input to create intelligent, personalized interactions, improving areas such as diagnostics, patient monitoring, mental health support, and chronic disease management (1, 2). Hybrid chatbots have demonstrated their potential to enhance chronic disease management, promote healthy behaviors, reduce hospital readmissions, and offer mental health support (6, 7). However, challenges such as trust, data security, system integration, and user experience persist (8, 9). Trust is a key barrier to patient adoption, as patients may hesitate to rely on chatbots for medical advice due to concerns about accuracy and data privacy (10, 11). Moreover, issues with integrating AI chatbots into existing healthcare systems hinder their ability to provide seamless patient care (12). Additionally, cultural and linguistic adaptability remains a concern, particularly in regions like India where language barriers affect communication (4). The objectives of the review include assessing the effectiveness of hybrid AI chatbots in healthcare, evaluating their role in improving patient engagement, reducing costs, enhancing diagnostic accuracy, and providing mental health support. It also aims to identify the barriers to adoption and propose solutions for overcoming challenges related to trust, data security, and user-friendliness (1, 2). The review will examine the role of AI chatbots in chronic disease management, mental health applications, and integration with healthcare IT systems like electronic health records (EHR), while also considering ethical, legal, and financial implications (3, 4). The novelty of the review lies in its comprehensive approach to addressing both the technological advancements and the challenges, particularly the cultural and emotional barriers that impact the acceptance and effectiveness of AI chatbots in global healthcare contexts. By focusing on emerging trends, including generative AI models, the review will guide future research and development in AI-driven healthcare solutions (17). Through a systematic methodology that includes eligibility criteria, review selection, data extraction, and synthesis, the study aims to provide actionable insights to improve the effectiveness, adoption, and integration of AI chatbots in healthcare, ultimately contributing to more efficient, patient-centered care (9).

Artificial intelligence (AI) is making substantial strides in revolutionizing healthcare, with AI-powered hybrid chatbots standing out as a key innovation. These chatbots combine the power of AI with human-like conversational abilities, creating a robust system capable of addressing multiple challenges within healthcare. By integrating generative AI, large language models (LLMs), and other AI technologies, these hybrid chatbots are improving patient care by offering real-time, personalized medical advice and support. The effectiveness of AI-driven chatbots is particularly notable in radiology, where AI models can analyze medical images with high precision, detect abnormalities, and assist in diagnosis and treatment planning. Hybrid chatbots, leveraging such technologies, can enhance patient interaction by providing tailored responses based on individual medical conditions or historical health data. This shift toward AI-powered solutions is seen to minimize human errors, reduce healthcare costs, and improve health outcomes for patients. However, the scalability and adaptability of these systems remain areas for improvement, particularly in real-world healthcare settings where patient needs vary widely (1, 2). The real-world applications and future potential of AI-powered hybrid chatbots in healthcare are promising. Case studies from various healthcare institutions, such as Sunway Medical Centre in Malaysia and clinics in Egypt, illustrate how these technologies have streamlined healthcare workflows and improved both patient outcomes and operational efficiency. Hybrid chatbots have shown promise in managing chronic diseases, supporting mental health care, and providing health education, where they offer continuous support and monitor patients remotely (9, 10). The technical mechanisms behind these chatbots rely on sophisticated algorithms like GPT-based models and BERT, which allow for the processing of patient queries and the generation of contextually relevant responses. These systems are also designed to integrate human expertise in complex cases, mitigating the risk of over-reliance on AI. Hybrid chatbots in healthcare have demonstrated significant benefits. They can reduce hospital readmissions by up to 25% for chronic conditions like diabetes and hypertension (7). AI chatbots increase patient interactions by 30%, improving care adherence (8), and reduce consultation wait times by approximately 15%, enhancing patient satisfaction (23). These findings highlight the positive impact of hybrid chatbots in improving patient care, engagement, and healthcare efficiency. Despite the promising capabilities, the widespread adoption of hybrid chatbots faces challenges, including infrastructure limitations, resistance from healthcare professionals, and concerns about data privacy and security. Overcoming these barriers through proper training, phased rollouts, and ensuring regulatory compliance will be crucial for unlocking the full potential of hybrid chatbots in transforming healthcare delivery (4, 30).

## Recommendations

### Invest in AI infrastructure

To enable AI-powered hybrid chatbots to function optimally, healthcare providers must prioritize investment in the necessary technological infrastructure. A robust infrastructure, which includes high-performance servers, cloud storage, and reliable data processing systems, is essential for ensuring the seamless operation of these advanced systems. These chatbots process vast amounts of patient data, and for them to function effectively, healthcare organizations must adopt scalable and secure IT frameworks that can handle the computational load. Moreover, integration with electronic health records (EHR) and other healthcare systems is crucial to ensure that chatbots can access up-to-date medical information and provide accurate, personalized recommendations. By investing in infrastructure, healthcare providers can also support future advancements in AI, ensuring that the systems remain adaptable and capable of evolving alongside technological trends (17).

### Focus on training and education

The successful integration of AI-powered hybrid chatbots into healthcare workflows depends largely on the preparedness of healthcare professionals. Providers should focus on comprehensive training programs that educate healthcare workers on how to use chatbots effectively, interpret their outputs, and address situations where human intervention is needed. This training should not only cover technical aspects, such as the functioning of AI systems and the handling of AI-generated recommendations but also emphasize the importance of patient communication and trust-building. For instance, chatbots may handle routine consultations or provide emotional support, but healthcare professionals should be equipped to interpret complex cases that require human expertise. Training will ensure that professionals use AI tools confidently, improving the overall efficiency of patient care and enhancing collaborative efforts between technology and human expertise (3, 18).

### Enhance user trust

For AI-powered hybrid chatbots to succeed, gaining the trust of patients is paramount. This can be achieved by ensuring data security, maintaining transparency, and adhering to ethical AI practices. Patients must feel confident that their personal health information is protected and handled responsibly. Clear communication about how AI systems collect, store, and process data, along with the implementation of robust security measures like encryption and secure data sharing protocols, is critical. Moreover, transparency in the decision-making processes of chatbots, such as how they generate recommendations based on patient data, will help mitigate concerns about the reliability and fairness of AI systems. Ethical AI practices, such as ensuring that chatbots are not biased and that they support patients equitably, are essential for fostering long-term trust in these technologies (4, 12).

### Standardize evaluation metrics

To ensure the effective use of AI-powered hybrid chatbots across healthcare systems, standardized evaluation metrics must be established. Consistent evaluation metrics will help healthcare providers assess the performance and impact of these technologies on patient outcomes, system efficiency, and cost-effectiveness. These metrics should be designed to measure various aspects of chatbot effectiveness, such as patient engagement, user satisfaction, and the accuracy of medical advice. Additionally, tracking metrics such as treatment adherence, patient recovery rates, and the reduction in healthcare costs due to remote monitoring and self-management is essential for gauging the broader impact of chatbot systems. Standardization will also allow for cross-comparison between different healthcare settings, enabling providers to identify best practices and areas for improvement (2).

### Expand research into long-term outcomes

While much research has focused on the immediate effectiveness of AI-powered hybrid chatbots, future studies should focus on their long-term impact on patient health outcomes, healthcare costs, and overall system efficiency. Research should explore how chatbot interventions influence chronic disease management, mental health care, and post-surgical recovery over extended periods. Long-term studies can reveal whether these technologies result in sustained improvements in patient well-being, reduction in emergency visits, and better management of health conditions. Furthermore, examining the economic benefits of AI-driven systems, including potential cost savings from reduced hospital admissions and fewer in-person consultations, will help policymakers and healthcare providers understand the full value of chatbot integration (7).

## Implications

### Optimizing healthcare delivery through AI-powered hybrid chatbots

AI-powered hybrid chatbots are revolutionizing healthcare by automating routine administrative tasks, such as appointment scheduling, patient inquiries, and symptom assessments. This reduces the administrative burden on healthcare providers, enabling them to focus more on complex medical procedures. Chatbots can also ensure patients receive timely responses, improving their overall engagement and satisfaction. By providing real-time assistance, these systems improve the efficiency of healthcare delivery, reduce wait times, and enhance the accessibility of care, particularly in underserved areas (2, 7). Ultimately, this can lead to better patient outcomes and streamlined operations.

### Addressing challenges in data security, privacy, and bias in AI integration

While AI chatbots hold great promise for healthcare, their integration poses several challenges, especially concerning data security, privacy, and potential biases. These systems handle sensitive patient data, requiring robust encryption and compliance with regulations such as HIPAA to protect patient confidentiality (4, 15). Furthermore, there is a risk that AI algorithms may reflect biases, leading to inequitable healthcare outcomes, especially for underrepresented populations (17). Addressing these issues is crucial for maintaining trust, ensuring legal compliance, and promoting fairness in AI-powered healthcare applications (22).

### Policy frameworks and legal implications of AI in healthcare

For AI-powered hybrid chatbots to be deployed responsibly in healthcare, governments and regulatory bodies must create clear policies and legal frameworks. This includes defining the roles and responsibilities of AI systems and human healthcare providers. Additionally, liability issues, particularly in cases of misdiagnosis or incorrect guidance by chatbots, need careful consideration to avoid legal complications (3, 30). Policymakers must also account for the broader impact of AI on healthcare accessibility, cost reduction, and service equity, especially in rural and remote regions, ensuring AI contributes positively to public health (18).

### Collaborative efforts for successful AI chatbot integration in healthcare

Successful integration of AI-powered chatbots in healthcare requires collaboration across various sectors. Healthcare providers, technology developers, and policymakers must work together to address operational challenges, ensure the systems meet clinical standards, and navigate regulatory requirements (13). This multidisciplinary approach is essential for overcoming hurdles such as bias in AI algorithms and ensuring that the technology enhances patient outcomes rather than replacing the human touch in healthcare. Continuous research, stakeholder engagement, and ethical considerations are vital for realizing the full potential of AI-powered hybrid chatbots in transforming healthcare globally (19).

## Limitations

While the integration of AI-powered hybrid chatbots into healthcare systems presents immense potential, several limitations must be considered for a comprehensive understanding of the challenges and feasibility.

### Technological constraints and adaptability

Despite the advancements in AI, the integration of hybrid chatbots faces limitations in adapting to diverse healthcare environments. Variations in healthcare system infrastructure, the complexity of medical conditions, and patient preferences may affect chatbot effectiveness (3, 18). Additionally, the use of chatbots for sensitive healthcare-related matters often requires technical enhancements to ensure they accurately interpret medical nuances (10).

### Ethical and legal concerns

The application of AI in healthcare raises ethical and legal issues, including privacy concerns and the potential for algorithmic bias. The handling of patient data must comply with stringent regulations such as GDPR and HIPAA, and the risk of unintentional bias in AI responses remains a challenge (3, 7). While AI models are trained on vast datasets, their ability to make contextually sensitive decisions, especially in varied cultural contexts, is still a topic of debate (21).

### Trust and user acceptance

The trust of patients and healthcare providers is crucial in the adoption of AI technologies. As identified in several studies, users’ reluctance to trust AI-based healthcare solutions due to concerns over data security, accountability, and the need for human intervention can limit the widespread use of hybrid chatbots in real-world settings (4, 11).

### Interoperability and integration

Hybrid chatbots often require seamless integration with existing healthcare systems. However, interoperability issues may arise, especially when linking AI chatbots with legacy medical records or other digital health technologies (7, 18). This may hinder the effectiveness and scalability of such systems.

### Scalability and cost

While AI chatbots can reduce operational costs, the initial setup and ongoing maintenance may be prohibitively expensive, particularly for smaller healthcare providers or those in developing regions (15). Additionally, scalability remains a concern, especially in areas with limited technological infrastructure (2, 14).

### Limited ability to handle complex scenarios

AI chatbots, though capable of handling routine queries, still struggle with complex medical cases that require human expertise. Many studies suggest that chatbots may lack the ability to address complicated clinical scenarios or provide comprehensive diagnostic assessments (13, 17).

### Cultural and linguistic barriers

AI chatbots’ performance can vary significantly based on cultural and linguistic factors. Despite progress in language models, chatbots often struggle with dialects, regional languages, and the subtleties of medical terminology, which can limit their effectiveness in diverse populations (8, 16).

These limitations indicate that while AI-powered hybrid chatbots hold promise, they must undergo further development to ensure their reliability, ethical compliance, and practical feasibility across different healthcare settings.

## Future research directions

The integration of AI-powered hybrid chatbots into healthcare presents a multitude of opportunities and challenges that warrant further exploration. Future research in this area can focus on several key dimensions, which could advance our understanding of their implications for healthcare providers, patients, and policymakers.

### User trust and adoption

Understanding the factors that influence patient trust in AI-powered chatbots is essential for their widespread adoption. Research by Chellasamy et al. (10) and Liou and Vo (4) highlights the importance of user trust in healthcare systems and its role in the acceptance of AI-based healthcare technologies. Future studies could explore how trust dynamics vary across different demographic groups and healthcare contexts, especially in regions with varying levels of technological adoption, such as developing countries (10).

### Ethical, legal, and technological considerations

As AI-powered hybrid chatbots are increasingly implemented in healthcare, ethical and legal implications need to be rigorously examined. Issues such as patient privacy, data security, and the potential for algorithmic bias must be addressed to ensure that these systems can be safely and effectively integrated into healthcare settings (2, 3). Future studies should investigate the ethical frameworks needed to guide AI deployment, as well as the role of regulations in safeguarding patient rights and enhancing chatbot effectiveness.

### Impact on healthcare outcomes

Investigating the direct impact of AI-powered hybrid chatbots on patient health outcomes remains a crucial area for future research. While chatbots have the potential to reduce hospital readmissions (7), further studies could quantify their impact on long-term health outcomes, particularly in managing chronic diseases (15). These studies should also assess the effectiveness of chatbots in improving patient engagement and satisfaction (1).

### Cross-cultural variations in healthcare chatbot usability

As demonstrated by Ekechi et al. (21) and Ramani et al. (16), user satisfaction with AI-infused chatbots can vary significantly between different countries. Future research should explore how cultural differences influence the usability and acceptance of AI healthcare chatbots, particularly in multicultural settings. Investigating how different linguistic, social, and cultural factors impact the design and performance of chatbots could help tailor solutions that are universally acceptable and efficient.

### Hybrid approaches for chatbot development

The use of hybrid chatbots, combining both AI-driven automation and human intervention, presents an area ripe for exploration. Research by (12) and Truong and Doan (25) underscores the importance of optimizing hybrid chatbot systems to enhance user experience. Future studies should focus on refining hybrid chatbot models to better balance automation and human touch, and investigate their potential in specialized healthcare domains, such as mental health (13).

### Scalability and sustainability

Future research should investigate the scalability of AI-powered hybrid chatbots across healthcare systems of various sizes. Studies could explore how these systems can be adapted to meet the needs of both large urban hospitals and smaller rural clinics (20). Additionally, examining the cost-effectiveness and long-term sustainability of these AI technologies in healthcare settings will be crucial for policymakers (8).

### Integration with other technologies

The potential for hybrid chatbots to work synergistically with other emerging technologies, such as IoT devices and blockchain (28), should be explored. Future studies could investigate how integrating AI chatbots with remote patient monitoring systems can improve real-time care delivery and disease management (17, 18).

### Personalization of healthcare interventions

Personalized healthcare interventions through AI chatbots could significantly improve the management of individual patient care (21). Research into AI-driven chatbots’ ability to tailor health advice based on patient history, preferences, and lifestyle could be a fruitful avenue for improving patient outcomes.

By addressing these research areas, future studies can provide deeper insights into the challenges and opportunities associated with AI-powered hybrid chatbots, ensuring that they contribute to more efficient, accessible, and patient-centered healthcare systems.

## Data Availability

The original contributions presented in the study are included in the article/supplementary material, further inquiries can be directed to the corresponding author/s.
